# A young female patient with multiple unilateral low-grade oncocytic renal tumors and angiomyolipoma: a case report and literature review

**DOI:** 10.1093/jscr/rjae125

**Published:** 2024-03-21

**Authors:** Yi Xu, Xuewen Jiang, Hui Meng

**Affiliations:** Department of Urology, Qilu Hospital of Shandong University, Jinan 250012, China; Department of Urology, Qilu Hospital of Shandong University, Jinan 250012, China; Department of Urology, Qilu Hospital of Shandong University, Jinan 250012, China

**Keywords:** low-grade eosinophilic renal tumors, renal angiomyolipoma, CK7, CD117

## Abstract

We identified a young female patient admitted for suspected renal malignancy. Partial nephrectomy was performed after imaging evaluation and discussion. Postoperative biopsy pathology reported multiple low-grade eosinophilic renal tumors (LOTs) with angiomyolipoma growth. After reviewing the data, we found that LOT was mostly solitary and occurred in middle-aged and elderly patients. This case is unique and we share it to improve the understanding of this disease.

## Introduction

There are many types of eosinophilic renal tumors, ranging from benign to malignant. Low-grade eosinophilic renal tumors (LOTs) have long been classified into the category of other eosinophilic tumors because of their morphological similarity to renal eosinophilic tumors (RO) and eosinophilic smoky cell carcinoma (eCRCC). To further understand their immunohistochemistry and genetics, LOTs were listed as a separate category for the first time in the 2022 classification by the World Health Organization (WHO). According to previous reports, LOTs usually occur in middle-aged and elderly patients and are primarily unilateral solitary tumors that do not grow with other tumors. We encountered a young female patient with multiple LOTs that grew simultaneously with renal angiomyolipoma (AML). We believe that the LOTs in this patient are unique, and report it here with the hope of improving our understanding of this tumor.

## Case report

A 29-year-old female was found to have abnormal echoes in her left kidney during a physical examination. The patient had no symptoms (frequent urination, urgency, dysuria, hematuria, low back pain, abdominal pain, fever, or fatigue). She had no history of chronic diseases such as hypertension and diabetes and was married with children. There was no history of food or drug allergies, smoking, alcohol consumption, or family history of hereditary diseases.

No obvious positive signs were found on general physical examination upon admission, and no signs of tuberous sclerosis were observed. No lumbar mass, percussion pain in the lumbar region, pressure pain in the ureteral tract, or abnormalities in the vulva were detected during specialized examination.

**Figure 1:**
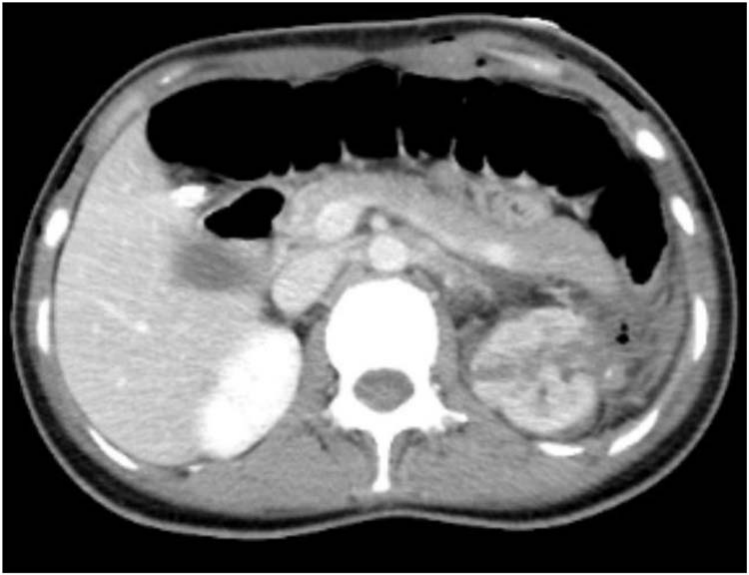
CT shows uneven density and disordered structure in the upper pole of the left kidney, with turbidity and increased density in the surrounding fat spaces.

To further clarify the diagnosis, an intensive computed tomography (CT) examination of the abdominopelvic region was performed, revealing a soft tissue mass and renal tumor in the left kidney, multiple small cysts in both kidneys, and multiple high-density foci in the pelvis, lumbosacral vertebral body, and adnexa (Fig. 1).

Following a comprehensive discussion under the multidisciplinary team model, a decision was made to proceed with surgical excision of the tumor. The preoperative examination revealed no contraindications to surgery. The procedure used involved da Vinci robot-assisted laparoscopic partial left nephrectomy. Intraoperatively, a tumor measuring ~3 × 4 cm was identified at the upper pole of the left kidney, and additional satellite tumors ~0.8 cm in size were detected near the primary lesion. All tumors were excised and sent for pathological examination. Rapid intraoperative pathological analysis indicated an eosinophilic cell tumor, prompting a localized excision. The surgery progressed smoothly, and postoperative recovery was satisfactory. The patient refused further genetic testing, and a 3-month post-discharge follow-up examination revealed no abnormalities in the surgical area. The patient is currently undergoing regular follow-up.

**Figure 2:**
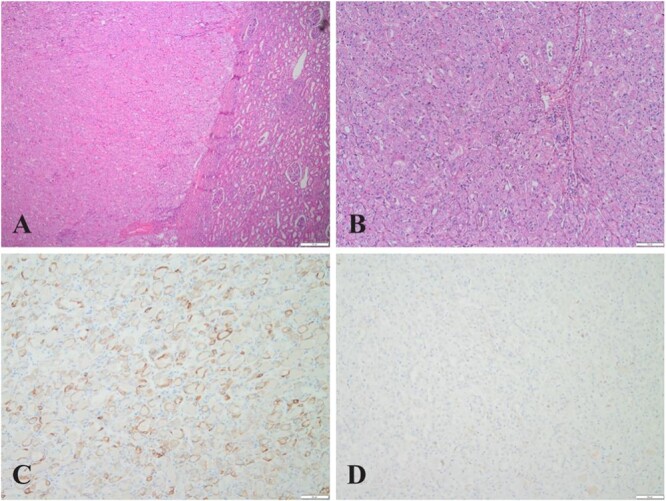
(A) (HE,×4). Eosinophilic cell tumor with well-defined tumor boundaries and acidophilic tumor cells in solid tubular, nested, and microcystic arrangements; (B) (HE,×10). Eosinophilic cell tumor, homogeneous eosinophilic or eosinophilic granular cytoplasm with no vacuoles in the cytoplasm, round to oval nuclei with some perinuclear halos and sparse binuclei with slender perinuclear vacuoles; (C&D) (×10). Immunohistochemistry showed tumor cells positive for CK7 but negative for CD117.

Four nodular tissue samples were examined postoperatively. Nodules 1 and 2 were identified as low-grade oncocytic renal tumors (LOTs). Microscopic examination revealed a distinct boundary between the tumor cells and normal renal tissue. The tumor cells exhibited a sheet-like arrangement with an eosinophilic cytoplasm (Fig. 2A); the nuclei were round or oval, with visible nucleoli. Some regions showed inconspicuous perinuclear halos (Fig. 2B). Immunohistochemical staining indicated positive expression of CK7 (Fig. 2C), partial PAX-8, and SDHB in the tumor cells, with no CD117 (Fig. 2D), RCC, CD10, or CA-IX expression. The Ki-67 proliferation index was ~1–2%.

**Figure 3:**
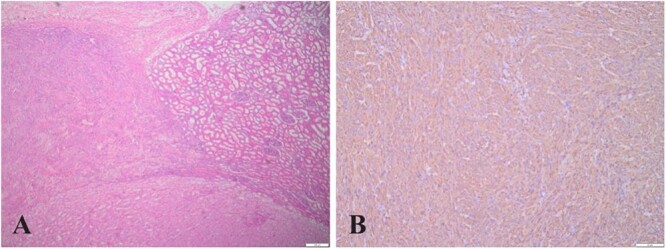
(A) (HE,×4). LOT, AML, and normal kidney tissue boundaries, where region a is AML, region b is normal kidney tissue, and region c is LOT; (B) (×10). Immunohistochemistry showing AML cells positive for SMA.

Nodule 3 contained both LOT and AML tissue. Microscopically, a clear demarcation was observed between the LOT, AML, and normal renal tissues (Fig. 3A). Immunohistochemistry revealed positive staining for smooth muscle actin (SMA) (Fig. 3B) in the AML as well as Desmin, HMB45, and Melan-A (occasionally), while CD117 was negative. Nodule 4 consisted exclusively of AML tissue.

## Discussion

Renal tumors that are eosinophilic, or that have eosinophilic characteristics, range from benign to malignant and often occur sporadically; multiple tumors have also been reported [1]. In 2019, Trpkov *et al.* reported 28 cases of tumors with low nuclear grade and eosinophilic cytoplasm that were CD117-negative/CK7-positive, naming them LOTs [2]. This term was then adopted as a new classification entity in the 2022 edition of the WHO classification of urological and male genital tumors [3].

In current reports, LOTs are sporadic solitary tumors that are more common in the elderly population. The average age of patients with LOTs is 63 years and they are more commonly found in women. The median tumor size is 2–3 cm, and there are typically no complications [2]. Microscopically, the tumors have round-to-oval-shaped nuclei and focal perinuclear halos, as well as areas of edema with scattered or irregularly distributed cells. Growing evidence suggests that they have characteristic immunohistochemical profiles (diffusely positive for CK7 and negative for CD117/KIT) [4].

Other immunohistochemical indicators in LOTs in addition to CK7 and CD117 were also studied. Chen *et al.* found that GATA3, E-cadherin, Pax-8, succinate dehydrogenase B (SDHB), and fumarate hydratase (FH) were all positive in five cases of LOT, while vimentin, carbonic anhydrase 9 (CA9), CD10, P504s, CK20, TFE3, TFEB, HMB45, ALK, and forkhead box protein I1 (FOXI1) were negative [1]. Williamson *et al.* found that GATA3 was positive in a study of 16 LOT tumors [5]. The immunohistochemical results of the patient presented here partially support this view. The detection of GATA3, PAX-8, SDHB, Pax-8, CD10, and other immune indicators may be significant for accurate clinical and differential diagnoses.

As molecular techniques such as next-generation sequencing are increasingly being used to evaluate LOTs, many case series have reported new findings. Ricci *et al.* found that MTOR, TCS1, and TSC2 mutations in the mTOR pathway were closely related to the pathogenesis of this tumor. Other mutated genes, including PIK3CA, NF2, and PTEN, were also identified that can act as both upstream and downstream effectors [6]. Williamson *et al.* also confirms the above conclusion [5]. Mutations in the mTOR pathway may play an important role in LOT diagnosis; however, these gene changes are nonspecific and are also common in several other eosinophilic tumors such as eosinophilic solid and cystic renal cell carcinoma (ESC RCC), renal eosinophilic vacuolated tumors (EVT), and renal cell carcinoma with vascular leiomyoma matrix (RCC FMS) [7]. For instance, Xia *et al.* recently found that LOTs, ESC RCC, EVT, and a group of similar tumors that do not fully meet these criteria all show TSC/mTOR mutations and have different RNA clustering expression profiles [8].

For LOTs, surgical resection is sufficient to achieve a satisfactory prognosis; no progression or metastasis has been reported with local resection or unilateral total nephrectomy. The key to the clinical diagnosis and treatment of this type of disease should therefore be the former. The prehospital diagnosis of the patient in this case was suspected to be malignant. The nature of the tumor was determined by rapid pathological examination during operation, and partial resection was performed. Clinical diagnosis requires knowledge of the pathological features of LOTs, timely puncture biopsy, or intraoperative pathological examination to avoid misdiagnosis and unnecessary treatment.

At present, English literature reports on the close growth of LOTs and AML are rare. In this case, the patient was only 29 years old but had multiple large tumors, which is relatively rare among reported cases. The patient refused to undergo genetic testing; genetic detection may have revealed a relationship between the LOTs and AML. An analysis of the diagnosis and treatment of these patients can enhance our understanding of LOTs.
